# UK Nutrition Research Partnership (NRP) workshop: Forum on advancing dietary intake assessment

**DOI:** 10.1111/nbu.12501

**Published:** 2021-06-04

**Authors:** Anne de la Hunty, Judith Buttriss, John Draper, Helen Roche, Georgia Levey, Ana Florescu, Naomi Penfold, Gary Frost

**Affiliations:** ^1^ British Nutrition Foundation London UK; ^2^ Institute of Biological Environmental and Rural Sciences Aberystwyth University Aberystwyth UK; ^3^ University College Dublin Dublin Ireland; ^4^ Imperial College London London UK; ^5^ Science Practice Ltd London UK

**Keywords:** biomarkers, dietary assessment methods, multi‐disciplinary, research, wearable technology

## Abstract

The development of better and more robust measures of dietary intake in free living situations was identified as a priority for advancing nutrition research by the Office of Strategic Coordination for Health Research (OSCHR) Review of Nutrition and Human Health Research in 2017. The UK Nutrition Research Partnership (NRP) sponsored a workshop on Dietary Intake Assessment methodology alongside its series of ‘Hot Topic’ workshops designed to accelerate progress in nutrition research by bringing together people from a range of different disciplines. The workshop on Dietary Intake Assessment methodology took place via Zoom over two half‐days in January 2021 and included 50 scientists from a wide range of disciplines. The problems with current methods of dietary assessment and how emerging technologies might address them were set out in pre‐recorded presentations and explored in panel discussions. Participants then worked in breakout groups to discuss and prioritise the research questions that should be addressed to best further the field and lead to improvements in dietary assessment methodology. Five priority research questions were selected. Participants were asked to brainstorm potential approaches for addressing them and were then asked to focus on one approach and develop it further. At the end of these sessions, participants presented their project ideas to the rest of the workshop and these will be reported back to the Medical Research Council. It is hoped that potential collaborative projects arising from these discussions will be taken forward in response to future funding calls.

## BACKGROUND

The UK Nutrition Research Partnership (UK NRP) for health and disease is a partnership between the Medical Research Council (MRC), the Biotechnology and Biological Sciences Research Council (BBSRC) and the National Institute for Health Research (NIHR), and is focussed on strengthening the UK science base in basic and translational nutritional research. It was formed as a direct implementation of the recommendations of the Office of Strategic Coordination for Health Research (OSCHR) Review of Nutrition and Human Health Research, published in 2017 (Buttriss, 2018; OSCHR, 2017). This review into the state of nutrition research in the UK concluded that, despite an impressive track record in nutrition research, the field was not reaching its full potential and that nutrition research studies were of ‘variable quality and intensity’ and that progress was slower and impact lower than might have been expected. One of the reasons the review gave for why progress was hampered was the lack of robust measures for what people actually eat in the free living situation and one of its recommendations, alongside better coordination of nutrition research, was ‘the development of standardised and validated objective measures of human dietary intake and human nutritional phenotyping with the aim of generating reliable data on patterns of diet and physical activity, nutrient status and individual variation in response’.

In October 2019, the UK NRP put out a call to accelerate progress by encouraging new thinking on important challenges in the nutrition field via the means of focussed workshops on specific nutrition ‘hot topics’. The aim of the workshops was to forge new links between different disciplines and to build research capacity by encouraging the formation of new multi‐disciplinary research teams able to address these challenges. The hope was to build a strong pipeline of ideas and collaborative projects that could be competitive for funding in the near future. A larger workshop on Dietary Intake Assessment, the subject of this article, was also sponsored by the UK NRP in addition to the ‘Hot Topic’ workshops.

The workshop on Advancing Dietary Intake Assessment was organised by Professor Gary Frost (Imperial College), Professor Helen Roche (University College, Dublin), Professor John Draper (University of Aberystwyth) and Professor Judy Buttriss (British Nutrition Foundation) with assistance from Dr Karen Finney (MRC), Georgia Levey (Imperial College) and Anne de la Hunty (British Nutrition Foundation). It was facilitated by Ana Florescu and Naomi Penfold from Science Practice. Two half‐day workshops took place via Zoom on 14th and 21st January 2021. The programme for the two days is shown in Box 1. The workshop was attended by 50 scientists from a wide range of disciplines, including those with a background in traditional methods of dietary assessment but also those with expertise in biomarkers, nutrigenomics, metabolomics, psychology, biostatistics, electronics and computer design, big data analytics, interactive systems design and artificial intelligence. The workshop was substantially over‐subscribed and those selected represented a cross section of experience with a good proportion of early career researchers attending as well as those with more extensive experience.

BOX 1 **UK Nutrition Research Partnership workshop: Forum on advancing dietary intake assessment**

**Table:** 

**Day 1**	
13:00	Welcome and Introduction by Professor John Mathers (Newcastle University)
13:25	Panel Discussion One: Challenges of Dietary Intakes Assessment
	Professor Nita Forouhi – Limitations of Dietary Intake Assessment
	Paul Finglas – Limitations of food composition/nutrient data
	Professor Lorraine Brennan – Challenges for the future
14:05	Breakout Groups: Generating Research Questions
14:40	Panel Discussion Two: Emerging Technologies
	Professor John Draper – Urine metabolomics and population sampling to develop biomarkers of food intake
	Dr Albert Koulman – Dried blood spot biomarkers and nutritional status
	Dr Breige McNulty – Improving individual reporting
	Dr Benny Lo – Wearable devices to measure food intake and eating behaviour
	Professor Diane O’Brien – Use of stable isotopes to assess food intake
15:15	Breakout Groups: Generating Research Questions
15:30	Breakout Groups: Prioritising Research Questions
16:00	Breakout Groups: Developing Ideas
16:40	Lightning talks
	Dr Nicola Pirastu – Understanding the role of diet on health using food liking and genetics
	Dr Susana Palma Duran – Lipidomics in nutrition and metabolic diseases
	Professor Jeff Brunstrom – Automated dietary assessments using a LArge‐capacity Remote Allocation (LARA) device
**Day 2**	
13:00	Welcome and recap
13:10	Panel Discussion Three: Funding Avenues
	Professor Brian Walker – A funding panel perspective on nutrition research
	Dr Karen Finney (MRC)
	Dr Louisa Jenkins (BBSRC)
13:30	Panel Discussion Four: Overcoming Challenges in Nutrition Research
	Dr Padma Maruvada – A view from the States
	Professor Gary Frost – Incorporating emerging tech in nutrition research
14:35	Breakout Groups: Develop and Refine Research Project Ideas
16:00	Present Project Ideas
16:30	Next Steps

## SESSION ONE: PROBLEMS WITH CURRENT METHODS OF DIETARY ASSESSMENT

The workshop began with an introduction from Professor John Mathers (Newcastle University) who set the context for the workshop and explained its aims and objectives. He began by pointing to the Global Burden of Disease study, which highlighted the impact of diet on health, and estimated that globally, 11 million deaths a year and over 250 million disability‐adjusted life years (DALYs) were due to poor diet (GBD, 2017). However, it was likely that this was an underestimate due to the problems associated with poor measurement of the diet and he reminded the participants of the recommendation by the OSCHR Review of the need for more robust measures of dietary assessment.

The measurement of what people eat is foundational to good nutrition research. This includes what people eat, how much is eaten and the composition of what is eaten. However, there are difficulties with measuring all three of these components leading to problems with both measurement error and uncertainty. In addition, asking people to record their diet can introduce changes to it so that they are not recording their habitual diet – an effect known as the Hawthorne effect. In fact, all self‐reported methods are susceptible to systematic bias and mis‐reporting. Professor Mathers outlined that there are potential solutions to these three problems of error, uncertainty and mis‐reporting through the use of digital technologies and the use of biomarkers. The aim of the workshop was to identify the most promising new technologies and approaches and to bring together people from a very diverse range of disciplines to spark new ideas and ways of addressing these longstanding problems.

The challenges of dietary intake assessment were elaborated in three pre‐recorded presentations by Professor Nita Forouhi (Nutritional epidemiology, MRC Epidemiology Unit, University of Cambridge), Paul Finglas (Food Databanks National Capability, Quadram Bioscience Institute, Norwich) and Professor Lorraine Brennan (Institute of Food and Health, University College, Dublin).

**Professor Nita Forouhi** discussed the complexity of diet and the many different components of it that might need to be measured depending on the particular research question. The question of whether a particular measurement method was fit‐for‐purpose needs to be considered since more precise and more objective measures such as the use of biomarkers or doubly labelled water were generally more burdensome and less easy to use than less precise and more subjective measures such as food frequency questionnaires and diet recalls. However, objective measures were less likely to be susceptible to mis‐reporting than subjective measures. The consequences of measurement error are that either true diet‐disease associations are masked as a result of within‐person random error or that findings are biased in either direction as a result of systematic over‐ or under‐reporting as a result of person‐specific biases arising from social or cultural desirability reasons. She ended with a summary of the challenges that need to be addressed: how to make dietary assessment simpler for individuals to recall/record their diets accurately; how to develop strategies to deal with measurement error; how to assess dietary intakes in ethnic minority groups in the UK or in global populations in low‐ and middle‐income countries; and how can objective (biomarker) measurement complement subjective assessments?

**Paul Finglas** discussed the limitations of food composition databases for dietary assessment methods. The UK nutrient database (CoFID) contains 3303 foods in 14 different food groups. Most of the data (90%) are based on analysis of the food, although some recipes, for example, might be based on calculations. The database does not contain much branded data and most of it is generic data based on sampling and analysis. The main limitations are the availability of data for specific branded foods and particularly for reformulated foods; the accuracy of the data, since they are average values; and the description of the foods in a way which makes it easy to match foods and to exchange data with other users. The use of both LanguaL and FoodEx2 to describe food in a more systematic way has allowed for better food identification and data exchange. Semi‐automatic or automatic computations using machine learning for food matching have also developed in recent years. The FoodEXplorer Tool on the EuroFIR website has compositional data on foods and nutrients for 38 countries which can be searched simultaneously. This increases the range of foods for which nutrient composition is available. The contribution of food composition data error to overall error in dietary intake estimates is relatively small compared to the error associated with measuring food intake and is around 10–15% at the most. Challenges that need to be addressed include better use of branded food data and greater use of machine learning to facilitate data acquisition and exchange.

**Professor Lorraine Brennan** discussed the challenges for developing the use of biomarkers of food intake. Biomarkers offer the potential for assessing dietary intake in an objective way since they do not depend on self‐report. However, they will not replace self‐reported data completely and the challenge is to combine objective methods with self‐reported methods. Biomarkers can be discovered either through acute intervention studies, where specific foods are fed to a group of people and then, through targeted metabolomics studies, particular biomarkers associated with these foods are identified or alternatively, by comparing the metabolomes of low and high consumers in a large cohort and then identifying the characteristic metabolites associated with high consumption of the food. In the first case, it is important to check that the findings in the small intervention study are replicated in larger cohorts and in the second approach, to assess the sensitivity and specificity of the biomarker in an independent study. She then went on to discuss how biomarkers should be validated and referred to the Food Biomarker Alliance (Foodball) recommendations which put forward eight criteria which should be addressed: plausibility, time–response, dose–response, reliability, reproducibility, performance, stability and robustness. The Foodball consortium also reviewed the current state of the art for biomarkers of different food groups such as legumes (Sri Harsha et al., 2018), apples, pears and stone fruits (Ulaszewska et al., 2018), berries and grapes (Ulaszewska et al., 2020), nuts and vegetable oils (Garcia‐Aloy et al., 2019), cereals foods (Landberg et al., 2019) and meat and seafood intake (Cuparencu et al., 2019). Once validated, biomarkers can be used: to measure adherence to a dietary intervention; as an objective measure of dietary intake; to calibrate and correct self‐reported data in a larger sample size; and in epidemiological studies as a surrogate for intake to model the relationship with health/disease parameters. She finished by setting out the challenges around developing the use of biomarkers. These are that there are very many unassigned features in metabolomics profiles and most metabolomics databases do not include biomarkers from foods; many putative biomarkers have not been validated or assessed for their specificity or their influences of other dietary factors; the use of biomarkers of dietary patterns is a huge area that has yet to be developed; it is not yet clear how many repeated measures are necessary to get a good representation of the habitual diet, and finally, the use of panels of biomarkers is another area that offers great potential that has yet to be harnessed.

### Panel discussion

During the discussion of the presentations, when asked what their field would look like in 10 years’ time, panellists described having rapid and accurate methods to record individuals’ intakes, having a library of biomarkers for different foods to combine with this data, making better use of up‐to‐date, branded data and having more accurate intake data through the combined use of self‐reported data with biomarker data. The length of time over which data needed to be collected to get a true reflection of someone's intake was discussed. While this depends on the particular research question, so that a single measurement could be sufficient for some questions, four days, including both a weekday and a weekend day, is a reasonable length of time which is not too burdensome for participants. The use of combinations of biomarkers also helps to improve the accuracy with which individuals can be classified into different categories of intake.

The question of whether it would ever be possible to have a single all‐purpose method that did not rely on combining different methodologies for assessing diet was discussed. It was felt that a one‐size‐fits‐all method would not be appropriate since it was important to use a method appropriate to the population of interest and the particular research question being addressed. It is more helpful to have a suite of tools and optimise them for the particular population and be able to combine appropriate ones to improve accuracy and precision. However, it should be possible to develop a decision matrix based on different filters and criteria which selects the most appropriate method for different research questions.

The use of biomarker data to correct self‐report data was thought to be useful for reducing mis‐reporting by different groups of the population, for example those who are overweight or obese, who represent a large proportion of the population. Combining self‐report data with biomarkers for specific foods, such as sugar, can give an estimate of the extent of over‐ or under‐reporting of that food. Biomarker data from a small sub‐sample can be used to calibrate self‐report data which can then be used to correct self‐report data in a larger sample. This would potentially reduce the amount of mis‐reporting in self‐report methods.

Other aspects of food intake, such as the time of day food is eaten, the context of eating and the timing of physical activity and sleep in relation to food intake are all factors that would ideally also be taken into account. Knowing the kinetics of a particular biomarker might give some information on when the food was eaten. The need for a broader dimension to understanding the relationships between diet and health, with involvement from other disciplines such as genetics and systems biology, is important to develop more personalised approaches.

## SESSION TWO: EMERGING TECHNOLOGIES ADDRESSING THESE PROBLEMS

**Professor John Draper** (University of Aberystwyth) outlined how urine metabolomics and population sampling could help to develop biomarkers of food intake. Potential urine biomarkers of food intake have now been described for a comprehensive range of foods/food groups in the UK diet which could provide objective measures of eating behaviour to help reduce inaccuracies that are common when self‐reporting diet, especially for groups for whom traditional methods are difficult. However, urine biomarkers need to be integrated with information on lipophilic biomarkers derived from analysis of dried blood spots to get a complete picture of nutritional status since lipophilic biomarkers are not found in urine which means that some foods are not covered by urinary biomarkers. Several samples are required to measure the habitual diet, and the particular urine sampling approach (24‐h, spot urines, morning fasting void etc) needs to be validated within new clinical trials designed specifically for biomarker technology validation. Nevertheless, urine biomarker technology used in conjunction with ‘slimmed down’ online diet recording tools should not only improve future data quality but should reduce burden on both researchers and participants as well as allow scope for scale‐up of studies.

**Dr Albert Koulman** (University of Cambridge) discussed the use of dried blood spot biomarkers for assessing nutritional status. Dried blood spots are much easier to collect than a blood sample as they can be done by participants at home and mailed to laboratories. They are therefore especially useful for populations such as infants where it is difficult to obtain blood samples. Nutritional status can be assessed for some nutrients using dried blood spots, but there are a number of limitations. For example, the volume of blood obtained is very small and, as the exact volume of blood is not known, the concentration of nutrients cannot be determined. Also, air exposure oxidises some nutrients such as vitamin C meaning they cannot be used to assess the nutritional status of some nutrients. Nevertheless, the reduction in costs and participant burden make dried blood spots suitable for use in large population surveys.

**Dr Breige McNulty** (University College, Dublin) summarised some recent improvements in methods of individual self‐reporting. New technologies such as web‐based applications or smart phone apps have been put forward as an alternative approach for collecting individual dietary intakes. Using these approaches to collecting individual intakes could potentially reduce the burden on researchers and participants, increase adherence, improve data analysis, while also reducing costs. However, how this type of technology can be used in different population groups, such as young children or the very old, still needs to be considered. In addition, other areas still to be explored include: whether these methods improve the level of mis‐reporting; whether they do reduce the burden on the participant; and finally, whether other, potentially useful data, such as details of the recipe used, are lost compared to some traditional methods.

**Dr Benny Lo** (Imperial College, London) outlined some developments in the use of wearable devices for measuring food intake and eating behaviour. He described recent work using wearable automatic cameras to count bites and to identify foods being eaten (Qiu et al., 2020). They have also developed a machine‐learning‐based approach to estimate portion size and showed that it was more accurate than visual estimation by dietitians () (unpublished data). This technology could also be useful in estimating individual intake when people are using shared plates (). One advantage of wearable technologies such as these is that they can potentially record food intake passively and might therefore give a more accurate record of habitual diet than self‐report methods, which potentially change the person's choice of food. Limitations of the technology include: privacy concerns since the cameras record images all the time; poor lighting, which affects the image quality; and user compliance with wearing the camera. In addition, more, well‐labelled data are required for training the artificial intelligence to improve the automatic identification of foods. The use of wearable devices, including their usability and feasibility, still needs to be validated in large‐scale studies and the ability of the automatic food identification system to determine micronutrient intake still needs to be assessed.

**Professor Diane O’Brien** (University of Alaska Fairbanks) explained how stable isotopes could be used to assess habitual intake of some foods. Stable isotope ratios of carbon 12 and 13 or nitrogen 14 and 15 are promising candidate biomarkers for fish and animal protein intake since the ratios for each vary in different biological systems (O’Brien, 2015). Although small, the variations in the ratios are highly predictable, and since the carbon and nitrogen in the body come solely from food, they can be used to ascertain the food source in the diet. For example, the nitrogen 15/14 ratio in fish is higher than that from other types of animal protein because the amount of nitrogen 15 accumulates up the food chain, and so the nitrogen 15/14 ratio can be used as a marker of fish intake. Stable isotope ratios integrate diet over several months, making them reliable measures of habitual intake. Analyses are automated, require very little sample, which could be hair or nail, and are robust to long‐term storage and freeze–thaw cycles, lending themselves well to studies using archived bio‐specimens. In addition, molecular stable isotope ratios are a new methodology that offers the potential to identify additional foods, including added sugars/sugar‐sweetened beverages (Johnson et al., 2021; Yun et al., 2020). However, some knowledge of a study population's diet is required for the application of these biomarkers since the ratio is associated with the most elevated source of the isotope in the diet.

### Panel discussion

During the discussion of these presentations, the question of how best to validate these emerging technologies was discussed. Various controlled feeding study designs are possible and different designs give different answers. There is a need to consider what would be the best study design to explore how to integrate technology with existing methods and either to allow for data capture from all the various new technologies in the same cohort or for an emerging method to be assessed across different cohorts.

The point was made that new technologies have already made massive changes in society and that they have the potential to hugely change this research area too. Citizen science offers one way of providing the data necessary for training the machine‐learning algorithms, provided the food images are accurately labelled. Integrating wearable cameras with other types of sensing technology, for example spectral imaging technologies or gas sensing technologies, which can measure biomarker concentrations in blood or expired air, would also give a fuller picture. Accessibility of the technology should not be too great an issue as the cost of these devices is not that high (£10–20) and they work in different populations such as children and teenagers and also in low‐income countries. However, they do need to be adapted for infants and children under 5 years. Acceptability and compliance are also high with people wearing the devices between 60 and 80% of the time as it is possible to detect when people are not wearing the device. Meta‐data associated with the images could also provide useful information in terms of where and when the food is eaten but these data often cannot be collected due to privacy concerns. The images would not be able to discriminate between different types of milk or sausage, for example, but because the camera is always recording, it is possible to capture nutrition information on labels and see food preparation processes that can help with more accurate identification of individual foods.

The use of biomarkers could potentially overcome problems with food composition variability, the variable effects of cooking and processing on food composition and differences in the bioavailability of nutrients from different foods but this approach requires further investigation before it can be in widespread use. Controlled feeding studies which look at the variability in the rate of appearance of the biomarker in blood or urine find that some biomarkers show little individual variability while others show a lot more. One aspect of biomarker validation, therefore, is to better understand individual variability in biomarker kinetics and to determine normal population ranges. Most biomarkers will probably be more useful for categorising consumption levels as high, medium or low than to be able to quantify people's actual intake of particular foods. While single biomarkers measuring single foods are available, for example proline betaine for oranges, in most cases a signature cluster of biomarkers would better discriminate a particular food or, alternatively, one or two biomarkers can together be a marker of a range of foods all containing a particular nutrient or bioactive compound, such as, for example, the surrogate biomarkers used to estimate flavan‐3‐ol intakes from tea, berries, pome fruits, cocoa‐derived products and nuts (Ottaviani et al., 2020).

One advantage of dried blood spots is that some analytes can be measured in dried blood spots that have been stored for up to 15 years. This raises the possibility of using historical samples in current research.

The need to bring in other disciplines was widely agreed, although it was recognised that the workshop had already brought in people from a broad range of different disciplines. Multi‐disciplinary collaborations were potentially very helpful to bring together people with a different set of assumptions and understandings of a problem, although it needs to be recognised that they could also be challenging. Experts in image analysis, sensing technology and big data could be useful if the problems of error and uncertainty are to be solved by collecting much greater quantities of data.

## SESSION THREE: A FUNDING PANEL PERSPECTIVE ON NUTRITION RESEARCH

**Professor Brian Walker**, the Chair of the MRC’s Population and Systems Medicine Board, set out the broad priorities for nutrition research from a funding panel perspective. In terms of MRC research priorities, many priority research areas are influenced by nutrition, although this is not spelt out. However, most nutrition research would fit into the Obesity, Nutrition and Health Behaviour strand of research which has an increasing focus on the behavioural determinants of health more broadly. The UKRI favours multi‐disciplinary approaches and it is important that nutrition research is integrated with other disciplines, and there is a connection between basic and mechanistic approaches through to clinical and population‐based approaches. Another important priority is capacity building and training the next generation of leaders in the field. Thirdly, partnership with industry is very much encouraged, despite the recognised challenges of working with industry. The BBSRC’s DRINC programme is a good example of that, albeit that it is no longer funding new research proposals.

The MRC and the BBSRC spend £60 million a year on nutrition‐related research and the MRC has increased funding for nutrition research in recent years. However, a lower proportion of nutrition research proposals compared to other disciplines are successful in achieving funding, and there are fewer large, sustainable, renewable grants awarded. Various problems with nutrition research proposals in general have been identified and these include the following: a focus on association rather than causation; the use of surrogate outcomes rather than hard endpoints; isolation from other relevant disciplines such as behavioural science or mechanisms; challenges with confounding; questions of feasibility; high cost; and generalisability beyond the specific group being tested. In conclusion, the sort of research that MRC is looking to fund would be cutting edge, multi‐disciplinary, intervention studies with clear outcomes, conducted in partnership with industry.

### Panel discussion

Dr Karen Finney, MRC Programme Manager for Nutrition and Gastroenterology, and Dr Louisa Jenkins, UKRI‐BBSCR Senior Portfolio Manager, were also part of the panel and said they were very happy to talk to participants individually after the workshop if people had questions about ideas for specific research proposals.

During the discussion, the question of where dietary assessment methodological research fitted into the MRC’s research priorities was raised, given that the focus of MRC funding is more on fundamental mechanisms and understanding rather than technology integration and method development. Professor Walker responded by saying it was necessary to really spell out in grant applications the reasons why method development was so important and the benefits that would be gained for other areas of research because this was not always apparent to referees. Given the priority the OSCHR Report had put on improving methods of dietary intake assessment, many workshop participants wanted to know whether there would be some sort of mechanism for ring‐fencing this area of research. UKRI representatives responded that there are strategic priority funds, although their size this year has not yet been agreed, and it is also possible to fund such research through response mode routes and these are large enough to cover multi‐disciplinary proposals. The main message from the funders was to talk to them at an early stage to be guided on what the best route for potential funding might be.

## SESSION FOUR: OVERCOMING CHALLENGES IN NUTRITION RESEARCH

**Dr Padma Maruvada** [Program Director: Division of Digestive Diseases and Nutrition, National Institute of Diabetes and Digestive and Kidney Disease (NIDDK)/NIH, US] set out what the US is doing in the area of biomarker research and nutrition research more generally. Nutrition research qualifies for nearly 4% of the NIH research budget and nearly half of the budget for the NIDDK, so around $0.5 billion. The US Department of Agriculture also funds nutrition research, and therefore, biomarker research falls across the two agencies. So to better align the efforts of both agencies and to address gaps in biomarker research, they have issued joint funding opportunity announcements and, over the last four years, have funded a substantial grant portfolio and created a critical mass of investigators interested in dietary biomarkers. Following a workshop on dietary biomarker development two years ago, they recognise that a concerted and coordinated effort is needed for biomarker development (Maruvada et al., 2020). As a result, they issued two funding opportunity announcements this year to identify and develop biomarkers of intake and compile a database of biomarkers available for the wider research community. They anticipate funding two to three dietary biomarker development centres as well as a coordinating centre to coordinate the entire programme. The aim is to develop biomarkers for foods and food groups across the USDA MyPlate guide. The programme will have three phases: identification of candidate biomarkers from controlled feeding studies of various food types and liquid chromatography–mass spectrometry (LC‐MS) metabolomics; evaluation of the candidate biomarkers for their performance and predictive power; validation of the candidate biomarkers against gold standard markers, such as 24‐h urinary sodium and dietary assessment methodologies.

She also outlined the broader nutrition research priorities of the NIH over the next decade (NIH, 2020). The unifying theme for their strategic plan is precision nutrition through four strategy goals: to spur discovery and innovation through foundational research; to investigate the role of dietary patterns and behaviours for optimal health; to define the role of nutrition across the lifespan for healthy development and ageing; and to reduce disease burden in clinical settings. Later this year, NIH will also be launching a common fund programme, the Nutrition for Precision Health Programme, to further knowledge in this area with the aim of developing algorithms able to predict individual responses to diet.

**Professor Gary Frost** (Imperial College, London) reflected on lessons that could be learned from previous progress with incorporating emerging technology into nutrition research. He began by emphasising that it was vital to recognise that there was a major problem with the traditional methods for assessing diet, and that until it was possible to have more confidence in our ability to measure habitual diet, it was difficult to progress other areas of nutrition research. This was recognised by funders and was highlighted in the OSCHR report as a priority area for research and therefore provides a real opportunity to move this area forward. He reminded participants that there had been progress in some areas: the myfood24 tool automated the process of inputting dietary intake data (Wark et al., 2018); a strategy for validating biomarkers had been developed (Lloyd et al., 2019); the use of metabolic phenotyping was being explored as a novel way of objectively assessing dietary patterns (Garcia‐Perez et al., 2017); and work was progressing on the use of artificial intelligence deep learning to recognise consumed foods as a way of passively monitoring dietary intake. These projects had been successful because they made a step change in exploiting the use of new technologies, they involved collaborative multi‐disciplinary teams and they attracted talented people from other fields.

### Panel discussion

During the discussion, Dr Maruvada confirmed that once established, the database on validated biomarkers would be publicly available and accessible to British and European researchers, similar to the existing metabolomics database. Opportunities for training and cross‐fertilisation between institutes in the two countries were also something that should be developed and encouraged.

## GENERATION OF PRIORITY RESEARCH QUESTIONS

After the two panel sessions on Day 1, workshop participants worked in breakout groups to discuss and prioritise the research questions that should be addressed to best further the field and lead to improvements in dietary assessment methodology. The six groups were each asked to prioritise three research questions. Understandably, there was a good deal of agreement between the different groups on what the priorities should be and the overall list of questions generated is shown below.

- How do we track actual energy intake and understand the relationship between actual and reported intake?
- How do we go from branded data with limited nutritional information (*i*.*e*. the limited data available on food labels compared to that in publicly available food composition databases) to extended nutritional data that contains additional information on micronutrient, vitamins and mineral (using data science, modelling or machine‐learning approaches in order to future‐proof dietary composition analysis)?
- How do we better identify the most viable (pragmatic, digital) technologies and analytical capabilities that exist currently in other scientific areas (*e*.*g*. engineering, computing, etc) to inform nutrition work?
- How do we best validate emerging technologies?
- How might we integrate dietary assessment technologies to measure habitual diet in individuals and populations?
- How might we prioritise context and also scale‐up and generalise dietary assessment methods? For example, can we work in partnership with big data from food industry partners?
- How might we integrate nutrition status biomarker and dietary intake biomarker data for long‐term assessment of nutrition?
- How might we capture/account for differences in bioavailability using a combination of subjective and objective techniques in various population groups (*e*.*g*. adolescents vs. older adults)?
- How can we combine self‐reported data and the information available from wearable data to get a better understanding of the accuracy of these methods?
- How do we combine the best bits of various different technologies and dietary assessment methods while maintaining and improving accuracy and precision?
- How can we use behavioural science to tap into why people are mis‐reporting and make dietary assessment less of a burden and also able to include data on emotions?
- How do we impute data to cope with missing data to address bias and error in self‐report data using machine learning?
- How do we measure dietary patterns and the interaction of different foods together (*e*.*g*. foods as whole or processed)?
- How to improve dietary intake assessment for people who are less able to use traditional methods or where other opportunities might exist (*e*.*g*. elderly people, school meals)?
- How to accurately assess what people consume when eating out?

Having generated the list of priority research questions, participants were asked to choose the research question that they would like to continue to work on during Day 2, to develop potential approaches for addressing this question. Five research questions were chosen by the workshop participants to work on. The five questions considered by the groups were as follows.

- How might we integrate dietary assessment technologies to measure habitual diet in individuals and populations?
- How do we combine the best bits of various different technologies and dietary assessment methods while maintaining and improving accuracy and precision?
- How might we integrate nutrition status biomarker and dietary intake biomarker data for long‐term assessment of nutrition?
- How might we improve dietary intake assessment for people who are less able to use traditional methods or where other opportunities might exists (*e*.*g*. elderly people, school meals)?
- How do we best validate emerging technologies?

The groups were asked to brainstorm potential approaches for addressing the research questions and then to focus on one and develop it further, to consider the methodology they would use, what makes the approach novel and innovative, what were the riskiest assumptions in their proposal, what resources would be required, and what skills and expertise they would require. At the end of these sessions, participants presented their project ideas to the rest of the workshop. A common experimental approach, identified by a number of groups, was a controlled study which evaluated and compared the results from wearable cameras, the measurement of various biomarkers and online self‐reporting in the same cohort.

## NEXT STEPS

The final stage of the workshop was a discussion on how best to take forward the ideas generated in the workshop. There was general agreement that the discussions had had been very productive and that it would be good to be able to continue them in some format. There was much interest in establishing a network of interested researchers, focussed on dietary assessment, to provide a forum to enable the exchange of ideas, to discuss potential collaborations and technique development and to advocate for more research in this area. This could, for example, be associated with the International Conference on Diet and Activity Methods (http://www.icdamportal.org). A further workshop to develop some of the project ideas in more detail was also popular, as was the idea to hold a series of topic‐focussed seminars to keep abreast of new developments.

In conclusion, Professor Frost thanked everyone for their hard work and enthusiastic participation and for recognising that it was time to put some serious thought into trying to solve the problems associated with dietary intake assessment. He also thanked the MRC for funding and supporting the workshop.
